# Serum aromatic *l*-amino acid decarboxylase activity as a biomarker for prodromal and manifest Parkinson's disease

**DOI:** 10.1016/j.ebiom.2026.106354

**Published:** 2026-07-09

**Authors:** Milan Beckers, Iris Kersten, Lukas Pavelka, H. Bea Kuiperij, Rejko Krüger, Bastiaan R. Bloem, Marcel M. Verbeek

**Affiliations:** a Department of Neurology, Donders Institute for Brain, Cognition and Behaviour, Radboud University Medical Center, 6525 GA Nijmegen, the Netherlands; b Radboudumc Centre of Expertise for Parkinson & Movement Disorders, 6525 GC Nijmegen, the Netherlands; c Transversal Translational Medicine, Luxembourg Institute of Health (LIH), 1445 Strassen, Luxembourg; d Translational Neuroscience, Luxembourg Centre for Systems Biomedicine (LCSB), University of Luxembourg, L-4362 Esch-sur-Alzette, Luxembourg; e Department of Neurology, Centre Hospitalier de Luxembourg (CHL), L-1210 Belair, Luxembourg; f Department of Human Genetics, Radboud University Medical Center, 6525 GA Nijmegen, the Netherlands

**Keywords:** Aromatic-L-amino-acid-decarboxylases, Dopa decarboxylase, Parkinson disease, REM sleep behaviour disorder, Biomarkers, Levodopa

## Abstract

**Background:**

Aromatic *l*-amino acid decarboxylase (AADC), or dopa decarboxylase (DDC), is a key enzyme in levodopa metabolism. Previous studies suggested that AADC protein levels may be increased in Parkinson's disease (PD), particularly if treated with levodopa in conjunction with a peripheral decarboxylase inhibitor (PDI).

**Methods:**

Serum AADC enzyme activity was measured by quantifying *ex vivo* conversion of levodopa to dopamine, and correlated to diagnosis, disease stage and clinical parameters in (i) *n* = 43 people with probable prodromal PD; (ii) *n* = 592 people with early PD (≤5 years since diagnosis), of whom *n* = 116 unmedicated; (iii) *n* = 48 medicated people with PD with longer disease duration (>5 years); and (iv) *n* = 74 non-parkinsonian controls.

**Findings:**

AADC activity was higher (*P* < 0.001) in probable prodromal PD (median 44.20 mU/L, IQR 34.30–58.60) and in manifest but unmedicated early PD (median 45.76, IQR 35.50–53.37) than in controls (median 35.09, IQR 26.92–44.49). Although AADC activity increased with disease duration (*P* < 0.001), there was no difference in AADC activity between probable prodromal and unmedicated manifest PD (*P* = 0.65). Use of dopaminergic medication increased AADC activity dose-dependently (median AADC in medicated PD: 110.09, IQR 85.44–145.82, *P <* 0.001).

**Interpretation:**

People with PD have higher serum AADC activity than controls, even in prodromal and unmedicated stages, suggesting a process driven by dopaminergic neurodegeneration. In medicated people with PD, AADC activity is markedly and dose-dependently increased. Results suggest that increased AADC activity could serve as an early biomarker for disease in unmedicated patients and that use of dopaminergic medication paradoxically induces AADC.

**Funding:**

This study was funded by Stichting Alkemade-Keuls. Underlying cohorts were funded by Verily Life Sciences LLC, Radboud University Medical Center, Radboud University, the city of Nijmegen, the Province of Gelderland, ParkinsonNederland, the Dutch Digestive Foundation, Stichting Woelse Waard, Stichting Alkemade-Keuls, the Luxembourg National Research Fund (FNR), and the European Union's Horizon 2020 research and innovation programme.

**Research in context**

**Evidence before this study**

Aromatic *l*-amino acid decarboxylase (AADC), or dopa decarboxylase, is a key enzyme in dopamine biosynthesis, and is also the rate-limiting step in levodopa drug therapy in Parkinson's disease (PD). Five recent studies using proximity extension assays demonstrated increased relative AADC protein levels in cerebrospinal fluid (CSF) in PD. Findings in plasma have been less consistent, with two studies demonstrating elevated relative plasma AADC protein in PD, and two studies finding elevated relative levels only in dopaminergically-treated patients, leading one group to hypothesise that the increase is solely driven by drug therapy. Research has thus far focused on relative protein levels in CSF and plasma, of which the practical applicability as a diagnostic biomarker is limited.

**Added value of this study**

In this study in a total of *n* = 757 human participants, using a readily-applicable enzyme activity assay in serum, we show that both unmedicated early PD and prodromal PD are associated with increased absolute serum AADC enzyme activity. We also demonstrate a paradoxical induction of serum AADC enzyme activity by levodopa use, and find indications of induced serum AADC activity correlating to a shorter duration of action of levodopa.

**Implications of all the available evidence**

The findings support the hypothesis that the increase in AADC activity is caused by the disease itself—possibly as a compensatory upregulation in response to a dopaminergic deficit—rather than merely being driven by dopaminergic therapy. This implies a possible use as a diagnostic biomarker for diseases associated with dopaminergic neurodegeneration—its translational potential thus reaching wider than only PD. The paradoxical induction of AADC enzyme activity and associated shorter duration of levodopa action suggest a possible secondary application as an explanatory biomarker for treatment response fluctuations. The blood-based assay using standard chemical laboratory supplies facilitates translation to a clinical setting.

## Introduction

**Fig. 1:**
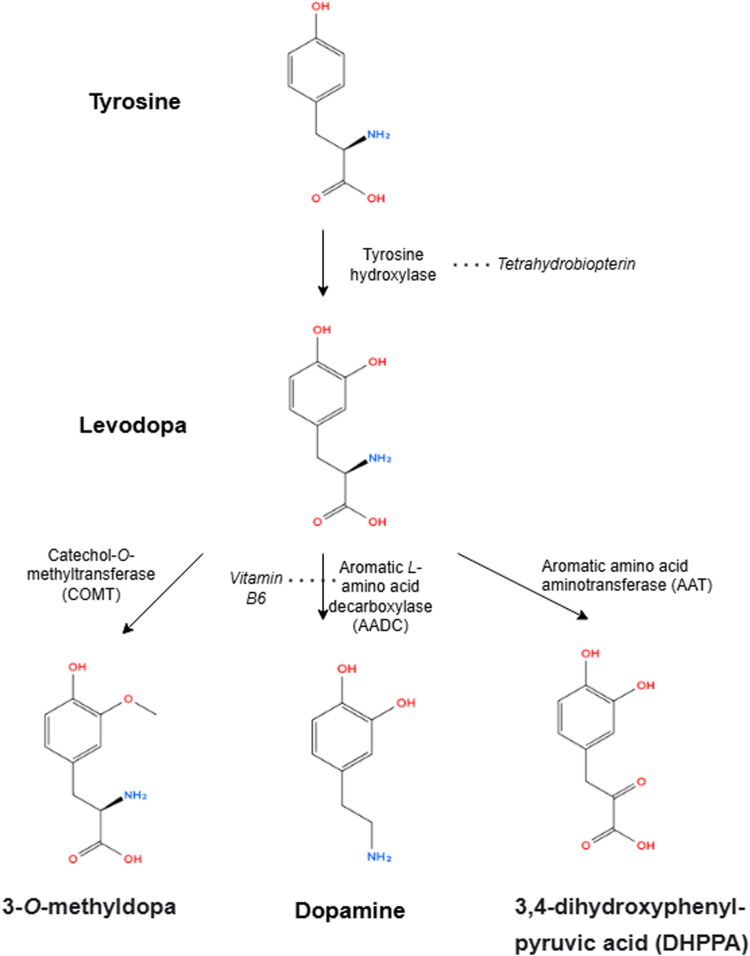
**Levodopa synthesis and metabolism.** Synthesis and metabolic pathways of levodopa. Bold: levodopa and its metabolic precursor and metabolites, normal: enzymes, italic: cofactors.

Aromatic *l*-amino acid decarboxylase (AADC),^1^ also referred to as dopa decarboxylase (DDC), is a key enzyme in levodopa metabolism. It decarboxylates levodopa to dopamine in the brain as well as in peripheral tissue and in the circulation, and so forms one of the main pathways of levodopa metabolisation (Fig. 1). Recent studies have consistently demonstrated increased AADC protein levels in CSF in Parkinson's disease (PD) in treated, untreated and even prodromal stages.^2^, ^3^, ^4^, ^5^, ^6^ Similar findings were reported in dementia with Lewy bodies (DLB), which shares important parts of its pathophysiology with PD,^3^^,^^5^^,^^7^ and also in other forms of atypical parkinsonism (AP) that have a different pathophysiology but have degeneration of dopaminergic neurons in common with PD.^5^^,^^6^ This suggests that the elevated AADC may be a compensatory mechanism to counter the dopaminergic deficit.^2^^,^^5^^,^^8^ Indeed, dopaminergic degeneration as demonstrated by DaT-SPECT imaging correlates with CSF AADC levels.^4^^,^^8^ AADC can therefore be of interest as a possible biomarker for diseases associated with dopaminergic degeneration.^9^

In contrast to the CSF findings, findings in plasma have been less consistent. Two studies found elevated plasma AADC protein in PD^4^^,^^5^ as well as in DLB and AP.^5^ Two other studies, however, only found elevated AADC protein levels in plasma of dopaminergically treated patients,^2^^,^^3^ leading one group to hypothesise that plasma AADC increase in Lewy-body disorders is solely caused by the use of dopaminergic medication.^3^

Besides its potential biomarker function, AADC enzyme activity is relevant for the efficacy of oral pharmacotherapy for PD. For oral levodopa to be effective in ameliorating PD symptoms, intact passage of levodopa across the blood–brain barrier is required. AADC enzyme activity is the rate-limiting step of dopamine synthesis in the brain in people with PD treated with levodopa.^10^ However, AADC enzyme activity in the periphery can lead to premature metabolisation of levodopa, decreasing the amount of levodopa reaching the brain and limiting its pharmacotherapeutic efficacy. Therefore, inhibition of AADC by peripheral decarboxylase inhibitors (PDIs, carbidopa and benserazide) is necessary to make oral levodopa a feasible and efficacious treatment in PD.^11^ The combination of levodopa with a PDI thus forms the cornerstone of pharmacological treatment of PD. However, previous research by our group showed that serum AADC enzyme activity is paradoxically induced in people with PD after long-term use of levodopa/PDI.^12^

The proximity extension assay (PEA) techniques used in recent studies measure relative levels of the AADC protein in plasma or CSF. Proteins are expressed as relative to the abundance of other proteins in the sample: normalised protein expression. This makes it challenging to extrapolate conclusions to absolute protein levels in individual persons. Moreover, protein levels do not necessarily correspond to enzyme activity. To overcome this limitation, an enzyme activity assay can be utilised. An important additional potential advantage of using an AADC enzyme activity assay is that this is a validated assay which is readily available for clinical use, stemming from its established use as a diagnostic for the genetic metabolic disorder AADC deficiency.^13^

In this study, we aimed to elucidate whether serum AADC enzyme activity is increased across the spectrum: from prodromal PD, through unmedicated early PD, to clinically manifest and dopaminergically-treated PD. Furthermore, we elaborate on whether increased AADC enzyme activity is associated with a diminished or aberrant efficacy of oral levodopa therapy.

## Methods

### Study design and population

This multicohort observational study comprised retrospective analyses of prospectively collected biosamples and clinical data, supplemented by a cross-sectional cohort specifically established for the present enzymatic biomarker study. Serum samples were sourced from five cohorts. (i) the Personalised Parkinson Project (PPP),^14^ a deeply-phenotyped cohort of people with early-stage (≤5 years of disease duration since diagnosis at inclusion) but mostly drug-treated PD; (ii) PPP de novo, a smaller cohort of very-early-stage (≤2 years, median 5 months) and untreated PD; (iii) the AADCTDC study, a cross-sectional study of people with more advanced PD (>5 years); (iv) individuals with polysomnography-proven isolated rapid eye movement sleep behaviour disorder (RBD) without clinically manifest PD (Luxembourg RBD Study); and (v) a heterogeneous cohort of non-levodopa-using controls who did not have a diagnosis of PD or another diagnosis associated with dopaminergic neurodegeneration (hereafter named ‘controls’). The control group was composed of individuals of whom leftover serum was available after venipuncture for clinical purposes and who did not object to the reuse of the samples for research purposes. All studies were performed at Radboudumc in the Netherlands, except for the Luxembourg RBD Study that was performed in Luxembourg. The cohort designs, their inclusion and exclusion criteria and their dates and locations of data collection can be found in Supplementary Box S1 as well as on ClinicalTrials.gov (reference numbers are provided in the ethics paragraph).

For the analysis, participants from several of these cohorts were re-grouped into analysis groups (AGs). Participants from the first two cohorts were pooled (which was valid from an analytical standpoint, as sample collection, processing and analysis were performed in exactly the same way in both cohorts) and classified into medically treated PD (AG1) and untreated PD (AG2). The advanced PD cohort was not pooled with the others, as the moment of sampling and the administered levodopa dose did not match that of the other cohorts (see below); this cohort was analysed separately (AG3) for parameters not available in the other cohorts (such as pertaining to delayed-ON and no-ON). Risk factors and clinical factors of participants in the Luxembourg RBD Study cohort were classified according to the 2019 Movement Disorder Society research criteria for prodromal Parkinson's disease^15^^,^^16^ and included in the ‘probable prodromal PD’ analysis group (AG4) if they exceeded the age-adjusted total likelihood ratio threshold for ‘probable prodromal PD’. The control cohort (AG5) was screened for factors unrelated to PD known to influence AADC enzyme activity, such as impaired renal and hepatic function, and use of medication with dopamine receptor blocking action. Despite the presence of such factors in a few individuals, this did not result in more outliers in AADC enzyme activity than in individuals without such factors present (Supplementary Table S1).

### Biosampling and AADC enzyme activity assay

Serum collection was done as previously published in the PPP study protocol.^14^

AADC enzyme activity was measured by quantifying the *ex vivo* conversion of levodopa to dopamine and expressed in mU/L. An adapted version of our previously developed assay^13^ was used for the quantification of AADC enzyme activity. The full procedure is described in Supplementary Box S2. The assay is specific for AADC enzyme activity, as the cofactor for AADC is added in excess (pyridoxal-5 phosphate), and essential cofactors for other levodopa-metabolising enzymes are absent from the assay buffers. For all cohorts, AADC enzyme activity was measured at Radboudumc, by the same laboratory technician. Sample haemolysis was rare, without unequal distribution over cohorts.

In each measurement batch of all cohorts the same pooled serum sample was included as quality control (QC), to check and correct for any differences between these measurements. These QC results were used to harmonise results across various batches of analyses. Therefore, we can reasonably exclude that differences in results were due to differences in the cohorts that were tested.

### Statistics

Statistical analysis was performed using SPSS version 31.0 (IBM). For testing of endogeneity—that is, a violation of the assumption of independence of statistical tests caused by an influence that the outcome variable exerts on the predictor variable—the EndoS macro for SPSS (version 03-01-2022)^17^ was used, and results reported as Hausman's F. For moderation models, the PROCESS macro for SPSS was used (version 4.2).^18^ Graphs were made using GraphPad Prism version 10.6.1 (GraphPad Software, LLC). Directed acyclic graphs were made using DAGitty version 3.1.

As per the predefined statistical plan, statistical analyses were done hierarchically. First, the primary outcome was analysed (difference in AADC enzyme activity according to diagnostic group, and diagnostic accuracy), then the secondary outcome (influence of dopaminergic medication on AADC enzyme activity), followed by the tertiary outcome (correlation between AADC enzyme activity and clinical response to levodopa), and finally exploratory analyses.

Extreme outliers (Z-score >3 or <−3) in AADC measurements were trimmed in order to obtain realistic interquartile ranges (IQRs); non-trimmed *P* values were reported in case outlier trimming inadvertently influenced significance.

Correlation analyses with continuous variables were performed using Spearman's rank correlation and reported as Spearman's rho (rho_sp_). Correlation analyses with nominal variables were performed using Chi square tests or Fisher's exact test, where applicable. Multiple linear and logistic regression analyses were employed to examine the relationship between predictors, covariates and outcome variables. Regression analysis results were reported as correlation coefficient (R) when a correlation was the outcome of interest, as coefficient of determination (R^2^) when predictive power was to be described, and as slope (β) if the influence of a specific (co)variate on the outcome parameter was to be described. For logistic regression models, 95% confidence intervals and odds ratios (OR) were reported. Bootstrapping (1000 samples) was employed in case of non-normality or heteroscedasticity. Regression tables were included in the Supplementary Materials.

Confounding was tested by the stepwise addition of potential confounders (as determined by a directed acyclic graph (DAG) based upon literature) to a hierarchical regression model as covariates. For the primary outcome, the following confounders were assessed: sex and age. For the secondary and tertiary outcomes, the following were assessed: sex, age, disease duration, levodopa-equivalent daily dose (LEDD), OFF-state Hoehn & Yahr stage, and OFF-state Movement Disorder Society-sponsored revision of the Unified Parkinson's Disease Rating Scale (MDS-UPDRS) part III score. If adding it to the model changed the regression coefficient of the predictor by >15%, the covariate remained in the statistical model and was adjusted for as a confounder; if not, it was removed from the model. Multicollinearity was assessed in regression analyses by calculating the variance inflation factor. None of the assessed possible confounders manifested multicollinearity.

Effect modification was tested by entering potentially relevant interaction terms (as per the DAG as described above) into the regression equation; if the interaction term was significant at the *P <* 0.05 level, the covariate was designated an effect modifier and analysed as part of a moderation model.

Nonparametric data were analysed using Mann–Whitney U and Kruskal–Wallis tests, and Wilcoxon signed rank test for repeated measures. Where applicable, Kruskal–Wallis effect size was given as η^2^. To examine the effect of covariates on nonparametric analyses, Quade's nonparametric ANCOVA was employed. Diagnostic performance was determined using receiver operating characteristic (ROC) analysis, and reported in terms of sensitivity, specificity, positive predictive value, negative predictive value, Brier score and accuracy; calibration plots were also generated.

### Ethics

All participants in the clinical cohorts had given their written informed consent for use of their biological material and clinical data for research purposes, in accordance with the Declaration of Helsinki. Permission by the ethics review boards for the studies was present (PPP: MREC Oost-Nederland reference 2016-2934, PPP de novo: Central Committee on Research Involving Human Subjects reference NL72631.091.20, AADCTDC: MREC Oost-Nederland reference 2022-15963, Luxembourg RBD Study: Comité National d'Ethique de Recherche reference 201407/13). The study protocol of the present study, making use of previously collected biosamples and data, was submitted to the MREC Oost-Nederland (reference 2022-15629) which concluded that ethical evaluation was not indicated. The Commissie Mensgebonden Onderzoek Radboudumc provided permission for the use of leftover biosamples for the control group in this study (reference 2016-3011). All clinical cohorts are registered on ClinicalTrials.gov (IDs NCT03364894, NCT04985539, NCT05558787, NCT05266872).

### Role of funders

Funders had no role in the study design, collection, analysis of the data, interpretation of the data, writing of the report or the decision to submit the paper for publication.

## Results

Demographics of the study population are shown in Table 1. The median estimated probability of prodromal PD (i.e. the probability, based upon a profile of risk and clinical factors, that a given participant had prodromal PD) in the full *n* = 50 RBD cohort, as determined using the MDS probable prodromal PD criteria, was 94%. In the final *n* = 43 group that met MDS probable prodromal PD criteria (AG4), this was 97%.

**Table 1:** Demographics.

	Controls (AG5)	Probable prodromal PD (AG4)	Early-stage PD without medication (AG2)	Early-stage PD with medication (AG1)	Advanced PD with medication (AG3)	*P*
*n*	74	43	116	476	48	N/A
Age (years)	59.0 (52.8–65.3)	67.1 (59.4–71.8)	65.0 (58.0–70.0)	64.0 (57.0–70.0)	67.3 (62.0–70.9)	***<0.001***^a^^,^^b^
Sex	*n* = 27 (36.5%) female	*n* = 13 (30%) female	*n* = 45 (38.8%) female	*n* = 192 (40.3%) female	*n* = 22 (45.8%) female	0.70^c^
Disease duration (months since diagnosis)	N/A	N/A	6.0 (3.0–14.0)	54.0 (39.0–69.0)	82.50 (71.25–102.0)	***<0.001***^a^^,^^d^
MDS-UPDRS III (OFF)	N/A	3 (1–7)	34 (27–41)	38 (29–48)	41.50 (29.50–47.75)	***<0.001***^a^^,^^e^
Medication use						
Levodopa	N/A	N/A	N/A	*n* = 461 (96.8%)	*n* = 48 (100%)	0.42^c^
Levodopa monotherapy				*n* = 256 (53.8%)	*n* = 13 (27%)	***<0.001***^c^
Levodopa + dopamine agonist				*n* = 185 (38.9%)	*n* = 30 (62.5%)	***<0.01***^c^
Levodopa + COMTi				*n* = 20 (4.2%)	*n* = 7 (14.6%)	***<0.01***^c^
Dopamine agonist monotherapy				*n* = 10 (2.1%)	*n* = 0 (0%)	0.62^c^
LEDD (mg)	N/A	N/A	N/A	642.6 (450.0–950.3)	892.5 (700.0–1256.3)	***<0.001***^f^
Hoehn & Yahr stage (OFF)	N/A	Mode 0 (range 0–0)	Mode 2 (range 1–3)	Mode 2 (range 1–4)	Mode 2.0 (range 1.0–3.0)	***<0.001***^c^^,^^g^

All values are median (IQR) unless otherwise specified. Abbreviations: AG: analysis group, COMTi: catechol-*O*-methyltransferase inhibitor, LEDD: levodopa-equivalent daily dose, MDS-UPDRS III: Movement Disorder Society-sponsored revision of the Unified Parkinson's Disease Rating Scale part III, N/A: not applicable.

a Kruskal–Wallis H.

b Post-hoc testing: significant difference is for controls vs. other groups, not between other groups.

c Chi-Square.

d Post-hoc testing: significant difference is between all 3 groups.

e Post-hoc testing: significant difference is between all groups except between AG1 and AG3.

f Mann–Whitney U.

g Post-hoc testing: significant difference is between all 4 groups.

In participants who used dopaminergic medication, serum samples were mostly taken in the medicated state. In the AADCTDC study, blood was drawn both in the practically-defined ‘OFF’ state (after 12 h of withholding dopaminergic medication)^19^ and a median of 87 min (IQR 78–95) after administration of a suprathreshold dose of levodopa/PDI. In the PPP cohort, blood samples were taken a median of 125 min (IQR 15–210) after administration of the participant's usual medication dose. The medication-to-venipuncture interval was greater in the PPP cohort than in the AADTDC study (*P <* 0.05). Controls were sampled a single time. The AADCTDC study consisted of a single visit, from which the samples were drawn. From the PPP study, if samples from multiple time points were available, the sample was chosen that was taken at the longest disease duration. From the Luxembourg RBD study, the baseline sample was used. Delayed sample processing (*n* = 17) did not affect AADC enzyme activity measurements (Supplementary Fig. S1).

### Primary outcome: AADC enzyme activity vs. diagnosis

**Fig. 2:**
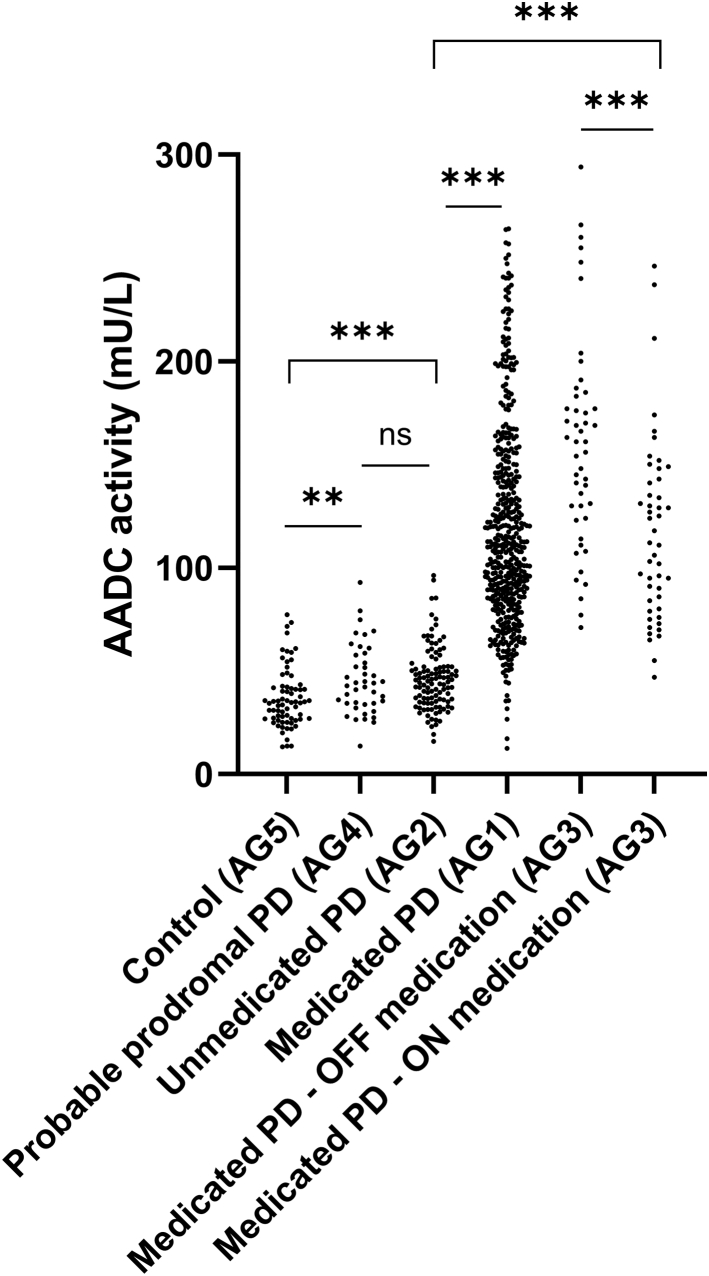
**Serum AADC enzyme activity in six groups.** Scatter-dot plots of serum AADC enzyme activity in the five analysis groups (AG1 *n* = 476, AG2 *n* = 116, AG3 *n* = 48, AG4 *n* = 43, AG5 *n* = 74). In the medicated advanced Parkinson's disease (AG3) group, measurement in the practically-defined OFF state (marked ‘OFF medication’, *n* = 48) and measurement after administration of a suprathreshold dose of levodopa/peripheral decarboxylase inhibitor (marked ‘ON medication’, *n* = 48) are displayed separately. Significance markers are for Kruskal–Wallis test with post-hoc inter-group comparisons. Significance, after Bonferroni adjustment for multiple testing, is noted by the number of asterisks: ∗∗*P* < 0.01, ∗∗∗*P* < 0.001, ns non-significant.

Median serum AADC enzyme activity was 35.09 mU/L (26.92–44.49) in controls (AG5), 44.20 mU/L (34.30–58.60) in probable prodromal PD (AG4) and 45.76 mU/L (35.50–53.37) in unmedicated PD (AG2) (Fig. 2).

Serum AADC enzyme activity was higher in probable prodromal PD than in controls (*P <* 0.01) As a diagnostic test for probable prodromal PD (vs. controls, AG4 vs. AG5), serum AADC enzyme activity had a moderate predictive power (area under the curve (AUC) 0.669, 95% CI 0.567–0.771, *P* = 0.003, Brier score 0.223, calibration plot provided as Supplementary Fig. S2). No confounding effect of sex or age was found.

**Fig. 3:**
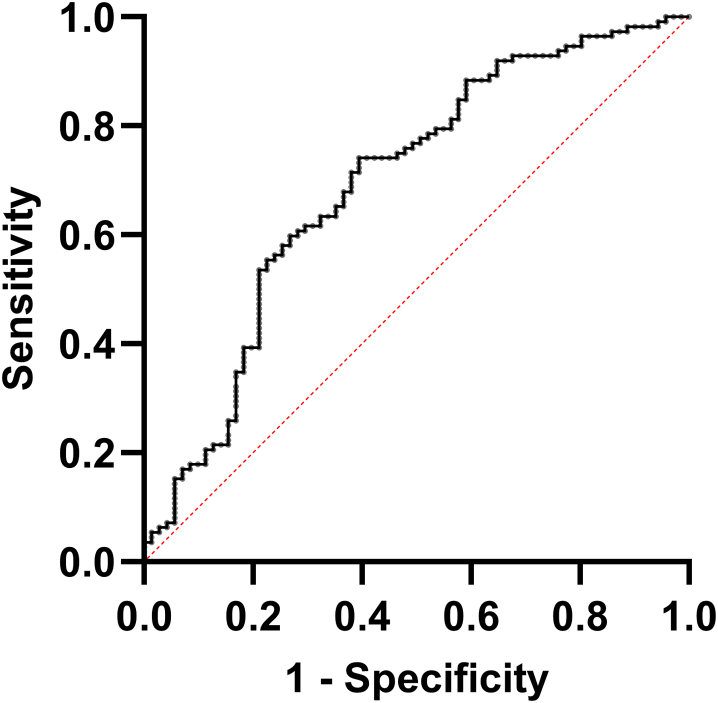
**Receiver operating characteristic (ROC) curve for serum AADC enzyme activity as a predictor for PD diagnosis.** Unmedicated, early Parkinson's disease (AG2, *n* = 116) versus controls (AG5, *n* = 74). Area under the curve 0.693; *P* < 0.001.

Likewise, serum AADC enzyme activity was higher in (clinically manifest, unmedicated) PD than in controls (AG2 vs. AG5, *P <* 0.001). As a diagnostic test, it had a moderate predictive power (AUC 0.693, 95% CI 0.612–0.774, *P <* 0.001, Brier score 0.223, Fig. 3, calibration plot provided as Supplementary Fig. S3). This was also not confounded by sex or age.

There was no significant difference in serum AADC enzyme activity between probable prodromal PD and unmedicated clinically manifest PD (*P* = 0.65, Fig. 2).

Various possible cut-off values for serum AADC enzyme activity and their diagnostic performance are provided in Supplementary Table S2.

### Secondary outcome: influence of dopaminergic medication on AADC enzyme activity

Median serum AADC enzyme activity in medicated PD (AG1) was 110.09 mU/L (85.44–145.82). After adjustment for disease duration, the use of dopaminergic medication predicted a higher serum AADC enzyme activity in people with PD (AG1+AG2, R^2^ 0.331, β 53.50, *P <* 0.001, Supplementary Table S3). The predictive value became stronger if only levodopa/PDI monotherapy users were considered (R^2^ 0.425, β 56.81, *P <* 0.001, Supplementary Table S4). Combination use of a catechol-*O*-methyltransferase (COMT) inhibitor with levodopa/PDI, in comparison to levodopa/PDI use without a COMT inhibitor, was weakly associated with an increase in serum AADC enzyme activity (R^2^ 0.086, β 28.08, *P* = 0.010, Supplementary Fig. S4, Supplementary Table S5).

The regression coefficient and significance were unchanged after adjusting for LEDD (which was not multicollinear with disease duration, variance inflation factor 1.136, rho_sp_ 0.343), implying that the correlation was not caused by increased levodopa bioavailability. Use of dopamine receptor agonist (DRA) monotherapy did not appear to be associated with a significantly different AADC enzyme activity compared to unmedicated PD (*P* = 0.491), although DRA monotherapy users comprised only 10 participants.

Age, sex, Hoehn & Yahr stage (in the OFF-medication state) and MDS-UPDRS part III score (in the ‘OFF’ state) were neither confounders nor effect modifiers for the relationship between dopaminergic medication use and AADC enzyme activity.

**Fig. 4:**
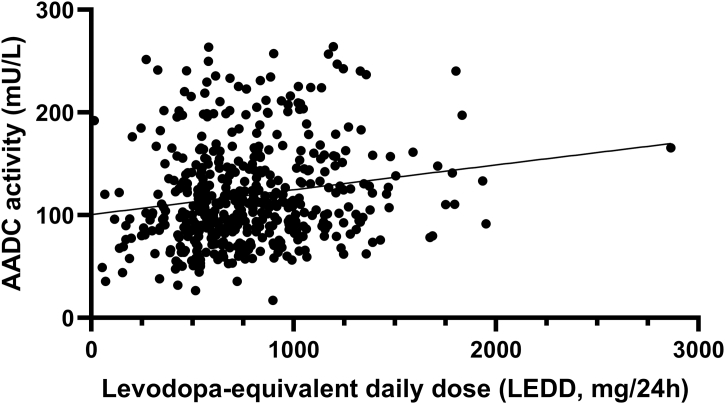
**Levodopa-equivalent daily dose (LEDD) adjusted for sex and disease duration vs. serum AADC enzyme activity.** Scatter plot with trend line of adjusted levodopa-equivalent daily dose (LEDD) versus serum AADC enzyme activity, in medicated early Parkinson's disease (AG1, *n* = 476). LEDD is adjusted for sex and disease duration and expressed at their modal (sex) or mean (disease duration) values.

Although the use of dopaminergic medication correlated to higher AADC enzyme activity independently of LEDD, higher LEDD weakly predicted higher AADC enzyme activity within the group of medicated patients (AG1), after adjustment for disease duration and sex (R^2^ 0.10, β 0.03, *P* < 0.001, Fig. 4, Supplementary Table S6). This relation was checked for endogeneity, as hypothetically, AADC enzyme activity could indirectly influence LEDD by decreasing levodopa bioavailability, necessitating higher medication doses. However, endogeneity was not present (Hausman's F 0.000, *P* = 0.99).

### Tertiary outcome: correlations between AADC enzyme activity and clinical response to levodopa

#### AADC enzyme activity vs. magnitude of levodopa response

The correlation between AADC enzyme activity and MDS-UPDRSIIIΔ_OFF-ON(%)_ was not determined in the medicated early PPP group (AG1) because of doubts of the validity of this particular score in this cohort, as explained in the Discussion section.

In the smaller advanced PD cohort (AG3), the MDS-UPDRS III data were more reliable, as 69% exhibited the expected MDS-UPDRSIIIΔ_OFF-ON_ of >30% and only 1 participant (2.1%) had a negative difference. In this cohort, no correlation between MDS-UPDRSIIIΔ_OFF-ON__%_ and serum AADC could be demonstrated, rho_sp_ being as low as −0.011 (*P* = 0.94) and a scatter plot of AADC enzyme activity versus MDS-UPDRSIIIΔ_OFF-ON%_ showing no hint of any correlation.

#### AADC enzyme activity vs. motor fluctuations

**Fig. 5:**
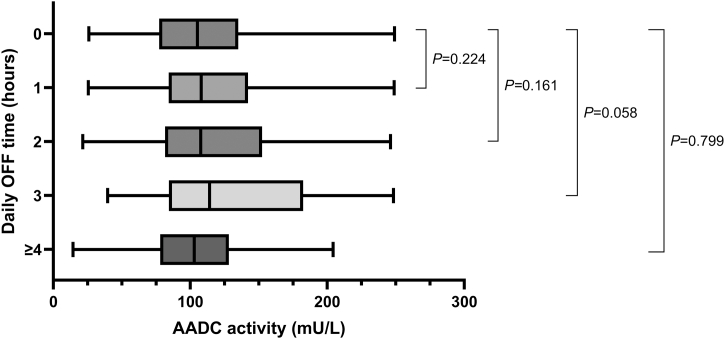
**AADC enzyme activity vs. daily OFF time.** Box plots of serum AADC enzyme activity in the medicated early Parkinson's disease group (AG1), grouped by daily OFF time (0 h *n* = 177, 1 h *n* = 73, 2 h *n* = 51, 3 h *n* = 25, ≥4 h *n* = 47, missing data *n* = 103). AADC activity is adjusted for disease duration, sex and LEDD and expressed at their mean (disease duration, LEDD) or modal (sex) values. *P* values are for multinomial logistic regression.

In medicated PD (AG1), after adjustment for disease duration, sex and LEDD, there was a non-significant trend towards a higher AADC enzyme activity correlating to more daily OFF time (Fig. 5). Only the difference between zero and three daily OFF hours approached significance (median adjusted AADC enzyme activity in people with zero OFF hours 104.46 mU/L, in people with three OFF hours 116.09 mU/L, difference 11%, *P* = 0.058, OR 1.008, 95% CI 1.000–1.017, Supplementary Table S7). The Hausman test (*P* = 0.62) did not indicate endogeneity in the relationship between AADC enzyme activity and daily OFF hours (MDS-UPDRS IV), albeit based on a relatively weak instrument (sex, Cragg–Donald F: 5.54). Because of this, endogeneity could not be entirely ruled out and caution is advised in the interpretation.

After adjustment for disease duration, sex and LEDD, there was a non-significant trend towards a small difference in AADC enzyme activity between a MDS-UPDRS item 4.5 (complexity of motor fluctuations) score of 1 (presence of motor fluctuations that are predictable) vs. a score of 0 (no motor fluctuations) (6% higher activity in the group with score 1, *P* = 0.065, OR 1.004, 95% CI 1.000–1.009, Supplementary Fig. S5, Supplementary Table S8). No (trends towards) significant differences were observed for scores of 2, 3 or 4 compared to a score of 0 in MDS-UPDRS item 4.5.

In the advanced PD cohort (AG3), correlation analysis was done into AADC enzyme activity versus time to ON (i.e. the measured time between levodopa administration and the moment at which participant indicated that the ‘ON’ state had been reached). There was no significant correlation between these variables (*P* = 0.58).

### Exploratory analyses

#### Demographics and disease characteristics vs. AADC enzyme activity

In clinically manifest early PD, AADC enzyme activity was higher in females than in males (Table 2). In medicated early PD, this effect of sex on AADC became more significant after adjustment for disease duration (defined as time since diagnosis) and LEDD, which acted as suppressors in this group. In medicated advanced PD, controls and probable prodromal PD, no sex difference in AADC enzyme activity was found.

**Table 2:** AADC enzyme activity versus sex.

	Male	Female	*P*
Controls (AG5)	34.7 (26.7–42.0)	34.3 (26.9–43.2)	0.97
Probable prodromal PD (AG4)	44.8 (35.5–61.5)	35.5 (27.7–52.7)	0.11
Unmedicated early PD (AG2)	41.5 (33.3–50.2)	48.24 (38.6–58.4)	***0.018***
Medicated PD early (AG1)	107.3 (83.0–141.1)	113.5 (89.1–156.5)	***<0.01***
Medicated advanced PD (AG3, OFF medication)	158.5 (107.8–203.3)	163.5 (128.5–183.5)	0.81
Medicated advanced PD (AG3, ON medication)	121.0 (78.3–156.3)	108.5 (87.5–134.3)	0.41
Total (without AG3)	83.9 (47.1–120.1)	93.5 (55.6–133.7)	***0.017***

AADC activity versus sex after trimming 10 outliers. Numbers are displayed in mU/L and as median (IQR), *P* values for medicated PD are for bootstrapped linear regression corrected for disease duration and levodopa-equivalent daily dose which act as suppressors in this group, other *P* values are for Mann–Whitney U. AG3 is not included in the total value as its moment of sampling is different from the other AGs.

AADC enzyme activity was not significantly correlated with age in any of the groups (combined groups rho_sp_ 0.004; *P* = 0.92). AADC enzyme activity did not significantly differ between the various Hoehn & Yahr stages, neither in unmedicated PD nor in medicated PD (combined groups η^2^ 0.003; *P* = 0.20).

In people with PD (combined group medicated + unmedicated, AG1+AG2), longer disease duration in months predicter higher AADC enzyme activity (R^2^ 0.131, β 0.467, *P* < 0.001, Supplementary Table S9), after adjustment for sex, levodopa use and LEDD.

#### Within-group analysis of probable prodromal PD

In participants with probable prodromal PD, there was no significant correlation between AADC enzyme activity and age, sex, RBD symptom duration, presence of motor symptoms, RBD severity, MDS-UPDRS part I, II or III scores, smell test scores, cognitive function, constipation, or family history of PD.

### AADC enzyme activity before vs. after intake of levodopa

**Fig. 6:**
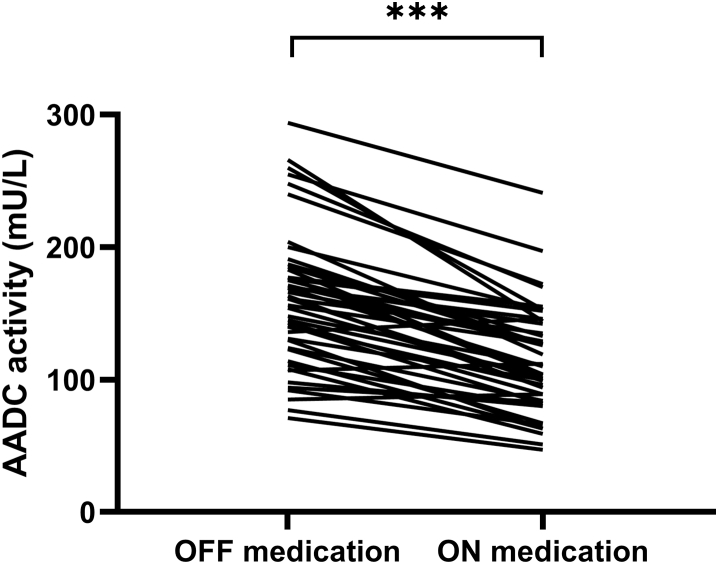
**Serum AADC enzyme activity before and after levodopa/PDI administration.** Spaghetti plot of serum AADC enzyme activity in advanced Parkinson's disease (AG3, *n* = 48 at both states). Each line represents the serum AADC activity of an individual participant, OFF medication (i.e. in the practically-defined OFF state) and ON medication (i.e. after administration of a suprathreshold dose of levodopa/peripheral decarboxylase inhibitor). ON values represent dose-adjusted enzyme activity standardised to the mean administered peripheral decarboxylase inhibitor dose. Significance marking is for the Wilcoxon signed rank test. ∗∗∗denotes significance at the *P* < 0.001 level.

In medicated advanced PD (AG3), serum AADC enzyme activity was tested both in the practically-defined OFF state (t_1_, after ≥12 h non-use of dopaminergic medication) and a median of 87 min (t_2_, range: 39–140) after administration of an oral suprathreshold dose of levodopa combined with a PDI in a 4:1 ratio. There was a strong correlation (rho_sp_ 0.901) between t_1_ and t_2_ activity. AADC enzyme activity at t_2_ was consistently decreased as compared to t_1_ (median 161.0 at t_1_ decreased to median 111.5 mU/L at t_2_, *P* < 0.001, Fig. 6). Nonetheless, AADC enzyme activity at t_2_ (lowered after PDI administration) was still higher than AADC enzyme activity in probable prodromal+unmedicated PD (111.5 mU/L vs. 44.8 mU/L, *P* < 0.001), as was AADC enzyme activity at t_1_ (161.0 vs. 44.8 mU/L, *P* < 0.001). Also, if medicated people with PD who had a high OFF-state AADC enzyme activity (defined as >184.50 mU/L, which is the 75th percentile at t_1_) were compared to medicated people with PD who had a lower AADC enzyme activity, the percentual decrease in AADC from t_1_ to t_2_ did not differ between the two (median_high_ 30.1%, median_low_ 29.4%, *P* = 0.6). Consequently, the resultant AADC enzyme activity at t_2_ (after levodopa/PDI administration) was higher in those with high OFF-state (t_1_) AADC enzyme activity than in those with lower OFF-state AADC enzyme activity (median_high_ 150.5 mU/L, median_low_ 95.5 mU/L, *P* < 0.001), also after adjustment for administered PDI dose.

## Discussion

The increased serum AADC enzyme activity in our cohorts of probable prodromal as well as early-stage, unmedicated people with PD suggests that this is driven by the disease itself, in contrast to the view expressed by others^2^^,^^3^ that elevated AADC in blood is solely driven by dopaminergic medication. Our present results support the hypothesis that neurodegeneration of dopaminergic neurons may trigger a compensatory upregulation of AADC production and activity.^2^^,^^5^^,^^8^ Our findings also suggest that dopaminergic neurodegeneration can be demonstrated using this biomarker in early disease stages and even in prodromal stages, when motor symptoms are not yet present. Our finding that higher serum AADC enzyme activity correlated to longer disease duration is in line with previous findings on AADC levels in plasma^2^^,^^3^ and further corroborates the hypothesis that it reflects dopaminergic degeneration, although in one previous study this correlation was not replicated.^4^

Our finding that serum AADC enzyme activity was elevated in early unmedicated and even (probable) prodromal disease contrasts with two studies that found normal plasma AADC protein levels in early disease.^2^^,^^3^ A possible explanation for this discrepancy may be that serum AADC *enzyme activity* may not necessarily correspond to plasma AADC *relative protein level*. Also, immunoassay-based analyses such as PEA are dependent upon binding of the specific antibody to AADC; there may, however, be different AADC proteoforms that do not all bind to the antibody.^4^ AADC enzyme activity analysis, conversely, quantifies *ex vivo* conversion of levodopa to dopamine and works independently of proteoforms. Furthermore, the studies reporting the absence of a correlation had adjusted their analysis for age,^2^^,^^3^ which may be inappropriate and may have resulted in type II error, as we have no data to support a correlation between age and AADC enzyme activity. In fact, in our cohort, the rho_sp_ of the correlation between age and AADC enzyme activity was as low as 0.004. The earlier papers provided no correlation analyses between age and plasma AADC, precluding assessment as to whether the adjustment was justified.

As described in previous papers,^12^^,^^20^ there is evidence to suggest that the increased AADC enzyme activity in levodopa/PDI users is caused by paradoxical induction of this enzyme. Several authors hypothesise^3^^,^^4^ that the increased AADC levels/activity observed in people with PD treated with levodopa may simply be a consequence of increased substrate (i.e. actual presence of levodopa in blood at a given time point) rather than a true induction (i.e. also demonstrable after levodopa levels have tapered off). Our findings are more in line with an actual induction. In our study with patients with advanced PD, we showed that AADC enzyme activity was also markedly increased (in treated patients as compared to unmedicated PD) when measured in serum drawn in the practically-defined OFF state, i.e. 12 or more hours after the last intake of dopaminergic medication. If the increased activity were a simple consequence of increased substrate, one would expect the AADC enzyme activity to be within normal limits when examined at such a time of absence of substrate. Given levodopa/PDI's short half-life of 90 min,^21^ a 12-h medication withdrawal is expected to result in a practical absence of substrate. Kinetic activation of AADC is rapid and short-lasting (hours)^22^; a substrate-induced activation would thus be expected to have subsided after 12 h.

The AUC of plasma levodopa concentration vs. time, as well as the maximum plasma concentration of exogenous levodopa, are normally higher in females then in males.^23^^,^^24^ Hypothetically, a lower AADC enzyme activity in females could be conceived as an explanation for this. However, in this study, we found the median serum AADC enzyme activity in females to be *higher* than in males, both in unmedicated and in medicated early PD. Differential AADC enzyme activity between the sexes is thus probably not the explanation for the different pharmacokinetics, and the underlying biological mechanism is not clear. If AADC enzyme activity were to be used as a diagnostic biomarker, a higher cut-off point for females may apply.

As a useful addition to the valuable work on the highly specific alpha-synuclein seed amplification assay (SAA),^25^ serum AADC enzyme activity may have added value as an extra biomarker to discriminate controls from patients with parkinsonisms. In contrast to SAA, one might expect serum AADC enzyme activity—being a marker for dopaminergic cell loss rather than for alpha-synuclein pathology—to be also increased in non-alpha-synucleinopathies, including specific genetic forms of PD such as PRKN or LRRK2. Combined use of SAA and serum AADC enzyme activity assay may thus well increase diagnostic accuracy. In addition, as AADC enzyme activity correlates to disease duration, it may be used as a marker for disease progression. Finally, in contrast to SAA, which needs specific equipment and is not available or reimbursed in many parts of the world, the AADC enzyme activity assay uses standard chemical laboratory supplies and could be easily accessible and scalable in a clinical setting. The AADC enzyme activity assay employed for this study is validated for clinical use. This makes it suitable for translational purposes, e.g. use in clinical trials and possible eventual use as a clinical biomarker.

The cut-off point for regarding the AADC enzyme activity assay as “positive” can be different, depending on the purpose of the test. For instance, for scientific purposes in which precision of the diagnosis is of paramount importance (e.g. disease-modifying trials), the low-sensitive but highly-specific 79 mU/L cut-off as described in previous research^12^ may well be suitable (sensitivity 7%, specificity 96%). Conversely, if serum AADC enzyme activity is used as a screening test, to be followed by a confirmatory test (such as dopaminergic imaging) in case of positivity, a lower cut-off point of 35 mU/L may be used to optimise sensitivity (sensitivity 76.7%, specificity 50%). In Supplementary Table S2, various possible cut-off points are given with their sensitivities and specificities.

Our findings showed that even in medicated people with PD who had an increased AADC enzyme activity in the practically-defined OFF state, administration of levodopa in conjunction with a peripheral decarboxylase inhibitor was able to temporarily suppress AADC enzyme activity, although the resulting AADC enzyme activity remained higher than in unmedicated individuals. This is consistent with the findings in an earlier study, which demonstrated that even in patients with a higher baseline AADC enzyme activity, administration of levodopa/PDI decreased AADC enzyme activity, stabilising 90 min after administration.^26^ However, we also found that within medicated people with PD, those with increased OFF-state AADC enzyme activity had a higher residual AADC enzyme activity after administration of levodopa/PDI, even after adjustment for PDI dose. This suggests that in people with PD with paradoxical AADC induction, PDIs are not able to suppress AADC enzyme activity to the same extent as in individuals who do not, or do to a lesser extent, have this induction. This presents a treatment dilemma, as the intuitive course of action would be to further increase the PDI dose to ensure adequate AADC inhibition. This may, however, paradoxically exacerbate the problem as AADC induction has been shown to be dose-dependent. Our results suggest that adding a COMT inhibitor to the drug regime may also further increase AADC activity. A possible explanation for this may be that inhibiting COMT diverts levodopa metabolism towards the AADC pathway, triggering further induction of the enzyme. This is a plausible mechanism, as the reverse has also been experimentally demonstrated: AADC inhibition diverts levodopa metabolism towards the COMT pathway and increases its metabolite 3-OMD.^27^ It should be noted that our interpretation of AADC activity being increased assumes that COMT inhibitors inhibit the metabolisation of levodopa to 3-OMD to the same extent as they inhibit the metabolisation of dopamine to 3-methoxytyramine. In the theoretical circumstance—for which we are not aware of any evidence—that the latter reaction is inhibited to a greater extent than the former, the resulting relative increase in dopamine may result in a shifted dopamine/levodopa ratio, creating the false impression of increased AADC activity.

For an aberrant response to levodopa caused by premature metabolisation, we have previously coined the term ‘peripheral levodopa resistance’.^20^ We previously hypothesised that serum AADC enzyme activity would also correlate to the magnitude and onset speed of levodopa action, i.e. that high AADC would correlate to a lower MDS-UPDRSIIIΔ_OFF-ON_ and to a longer time to onset (delayed-ON and no-ON), respectively. This now appears to not be the case. Rather, analogous to COMT activity, the activity of AADC correlates with a reduced duration of action of levodopa. An AADC-mediated higher rate of metabolisation may cause levodopa blood concentrations to fall below therapeutic levels sooner than desired, leading to increased occurrence of the OFF phenomenon. The relatively weak correlation between serum AADC activity and daily OFF duration suggests, nonetheless, that increased serum AADC activity is unlikely to be the sole explanation for the phenomenon of ‘peripheral levodopa resistance’ and that other mechanisms—e.g. premature levodopa metabolisation by gut microbiota—may also play a role.^28^

The trend towards higher serum AADC enzyme activity correlating to longer daily OFF duration (*P* = 0.058) as well as occurrence of motor fluctuations (*P* = 0.065) suggests a possible detrimental impact of paradoxically induced AADC enzyme activity on the efficacy of (oral) levodopa therapy. The serum AADC enzyme activity assay may therefore also be useful as an explanatory biomarker for certain cases of diminished clinical response to levodopa. In those circumstances, timing of venipuncture for AADC enzyme activity analysis is of paramount importance, given the stark difference between serum AADC enzyme activity in the OFF state and serum AADC enzyme activity after the intake of levodopa/PDI. While this study confirms that PDIs are still able to significantly lower AADC enzyme activity, even in individuals with increased AADC enzyme activity, the resulting AADC enzyme activity remains higher than in both untreated people with PD and people with PD with lower baseline AADC enzyme activity. This suggest that conventional treatment with PDIs may fall short of what is needed to effectively suppress AADC enzyme activity in these individuals.

This study has several strengths: the use of large and deeply-phenotyped cohorts from different stages of the disease, and the use of absolute enzyme activity rather than relative protein levels, which circumvents the limitations of PEA.

Limitations include the relatively short disease duration of many participants, 4 years and 2 months being the median disease duration of the pooled groups AG1+AG2+AG3. This also potentially influences the reliability of correlation of AADC enzyme activity with disease severity (Hoehn & Yahr stage). A further limitation is that the correlation between AADC enzyme activity and MDS-UPDRSIIIΔ_OFF-ON_ was not determined in the PPP cohort. This is because, in that cohort, the validity of the MDS-UPDRSIIIΔ_OFF-ON_ was questionable. Based upon the MDS diagnostic criteria for PD,^29^ in which an excellent levodopa response (MDS-UPDRSIIIΔ_OFF-ON_ >30%) is a supportive criterion for the diagnosis, and a poor levodopa response is an exclusion criterion, the large majority of a cohort of patients with early-stage PD would be expected to have a MDS-UPDRSIIIΔ_OFF-ON_ >30%. However, in the PPP cohort, the median MDS-UPDRS III improvement in the ON state was only 15.4% and in fact, 11.3% (*n* = 54) of the participants in the medicated PD group had a *negative* difference, meaning that they scored worse in the ON state than in the OFF state. Because of these results, probably caused by participants' fatigue (noting that the ON-state assessments always followed the OFF-state assessments, during an intensive day of testing) and which cannot be extrapolated to the larger PD population, any attempt to correlate these numbers to AADC enzyme activity would be pointless.

On the (pre-)analytic level, possible limitations include that it cannot be fully excluded that a) variations in sample collection, handling and (pre)processing influenced the inter-cohort differences in results; b) the assay may not purely reflect AADC activity but may be influenced by other pathways of levodopa and dopamine metabolism (such as by oxidation or through COMT, dopamine-β-hydroxylase and monoamine oxidase). It would be expected, however, that this would equally affect measurements across samples and cohorts. Furthermore, pre-analytic factors were controlled for by maintaining strict standard operating procedures, and the assay was optimised for AADC, lacking essential cofactors for other enzymatic reactions. The absence of a difference in AADC activity results between same-day processed samples versus delayed-processed samples demonstrates that the assay is quite robust to this kind of (pre-)analytic variation.

Another limitation of the study pertains to the use of blood as the medium for the AADC enzyme activity measurement. The AADC gene is notably more expressed in the small intestine than in peripheral blood^30^ and the same probably applies to AADC's action.^31^ With regard to AADC enzyme activity as a cause of/biomarker for decreased levodopa efficacy, this begs the question whether serum is the most valid point of sampling. In future research, it would be interesting to correlate AADC enzyme activity in serum to AADC enzyme activity in the small intestine (e.g. by endoscopic biopsy).

A further limitation has its bearings in methodology and statistics. In cohorts of people with PD who use dopaminergic medication, *endogeneity* forms a major obstacle in analysing the relationship between AADC enzyme activity, levodopa dose and clinical outcomes. Several studies have proposed or concluded that a higher levodopa dose results in higher AADC enzyme activity. However, this relationship might well be endogenous, i.e.: a higher AADC enzyme activity leads to lower levodopa bioavailability, thereby decreasing PD symptom control, prompting the treating physician to increase the levodopa dose. In this conceptual model, the predictor variable levodopa dose is not exogenous (i.e. independent of the outcome variable), violating the assumption of independence that is a condition for many of the employed statistical techniques. To overcome this, either a prospective study design is necessary in which levodopa doses are standardised and not adjusted to individual participants' symptoms (which would raise ethical objections) or a design in which multiple so-called instrumental variables are included: variables that—to some extent—predict an independent variable (e.g. AADC enzyme activity) without being correlated to the outcome variable (e.g. clinical response to levodopa). In addition to sex, other hypothetical instrumental variables for future studies may be alpha-synuclein SAA results and quantified DaT-SPECT binding,^8^ or perhaps genetic polymorphisms that predispose for AADC variability.^32^, ^33^, ^34^

In the five-phase framework for biomarker development in the Geneva roadmap,^35^ originally developed for Alzheimer's disease but also useful for PD, serum AADC enzyme activity is currently in phase 3. In order for it to progress to phases 4 (real-world performance) and 5 (implementation), more longitudinal and prospective validation is necessary to ascertain the value of serum AADC as a biomarker, both as a diagnostic biomarker and as a biomarker for premature levodopa metabolisation. Other future research may include correlation of AADC enzyme activity to AADC protein levels, using different biosamples for AADC enzyme activity analysis (e.g. small-intestinal biopsy), correlation of AADC enzyme activity/protein to other potential biomarkers such as alpha-synuclein SAA, CSF AADC (enzyme activity or protein levels) and further examination of the relationship between AADC enzyme activity and levodopa response in a prospective cohort with fixed doses/instrumental variables. Ideally, such a prospective cohort would also include plasma pharmacokinetic profiling of levodopa to ascertain the three-way correlation between AADC enzyme activity, levodopa pharmacokinetics and clinical response in these individuals.

In conclusion, people with PD have significantly higher serum AADC enzyme activity than non-PD controls, even in prodromal and unmedicated stages, suggesting a process driven by dopaminergic neurodegeneration. The results suggest that serum AADC enzyme activity could serve as a diagnostic biomarker. In medicated patients, AADC enzyme activity is markedly and dose-dependently increased. Despite this paradoxical induction, peripheral decarboxylase inhibitors can still suppress AADC enzyme activity, but the resultant activity remains higher than with less or no such induction. The paradoxical induction of AADC enzyme activity and associated shorter duration of levodopa action suggest a possible secondary application as an explanatory biomarker for treatment response fluctuations. The blood-based assay using standard chemical laboratory supplies facilitates translation to a clinical setting.

## Contributors

MB: Data curation, Formal analysis, Writing – original draft, Visualisation, Project administration; IK: Resources, Investigation; LP: Resources, Investigation, Writing – review & editing; HBK: Writing – review & editing; RK: Resources, Investigation, Writing – review & editing, Conceptualisation, Funding acquisition; BRB: Resources, Writing – review & editing, Conceptualisation, Funding acquisition, Supervision; MMV: Resources, Writing – review & editing, Conceptualisation, Funding acquisition, Supervision. MB and MMV accessed and verified the underlying data. All authors have read and approved the final version of the manuscript.

## Data sharing statement

Anonymised data that support the findings of this study are available to interested academic investigators from the corresponding author (milan.beckers@radboudumc.nl) upon reasonable request, after approval of a proposal and with a signed data access agreement.

## Declaration of interests

MB was a recipient of the Edmond J. Safra Fellowship in Movement Disorders. He received consultancy fees (paid to the institute) from AbbVie Netherlands and Merz Pharmaceuticals and received research funding (paid to the institute) from ParkinsonNederland, the Dutch Digestive Foundation, Stichting Woelse Waard, AbbVie and Ever Neuro Pharma GmbH. LP was a recipient of the Thiemann Fellowship 2024 by the Thiemann Foundation (Germany). RK serves as Editorial Board Member of the European Journal of Clinical Investigation, the Journal of Parkinsonism and Related Disorders and the Journal of Neural Transmission. He is receiving or has received research grants from the Fonds National de Recherche (FNR) Luxembourg, Fondation Veuve-Metz-Tesch Luxembourg, the Leir Foundation, the Michael J. Fox Foundation for Parkinsonʼs Research (MJFF), the European Institute of Innovation and Technology (EIT Health), the Innovative Medicines Initiative (IMI) of the European Union and the European pharmaceutical industry, and the European Union's Horizon 2020 and Horizon Europe research and innovation programs. He received speaker's honoraria and/or travel grants from Abbvie, Bial, Desitin, Medtronic and Zambon. He is participating or has participated as PI or site-PI for industry sponsored clinical trials without receiving personal honoraria. BRB serves as the co-Editor in Chief for the Journal of Parkinson's disease, serves on the editorial board of Practical Neurology and Digital Biomarkers, has received fees from serving on the scientific advisory board for the Critical Path Institute, Gyenno Science, MedRhythms, UCB, Kyowa Kirin and Zambon (paid to the Institute), has received fees for speaking at conferences from AbbVie, Bial, Biogen, GE Healthcare, Oruen, Roche, UCB and Zambon (paid to the Institute), and has received research support from Biogen, Cure Parkinson's, Davis Phinney Foundation, Edmond J. Safra Foundation, Fred Foundation, Gatsby Foundation, Hersenstichting Nederland, Horizon 2020, IRLAB Therapeutics, Maag Lever Darm Stichting, Michael J Fox Foundation, Ministry of Agriculture, Ministry of Economic Affairs & Climate Policy, Ministry of Health, Welfare and Sport, Netherlands Organisation for Scientific Research (ZonMw), Not Impossible, Parkinson Vereniging, Parkinson's Foundation, Parkinson's UK, Stichting Alkemade-Keuls, Stichting Parkinson NL, Stichting Woelse Waard, Health Holland/Topsector Life Sciences and Health, UCB, Verily Life Sciences, Roche and Zambon. MMV is supported by research grants from ParkinsonNL (project P2021-18), the Dutch Digestive Foundation (project WOO 21-05), Stichting Woelse Waard and Stichting Alkemade-Keuls, ZonMW – Dementia program (10510032120006 and 10510032120003), Alzheimer Nederland (WE03-2023-11) and the Radboud Fonds. The Radboudumc Centre of Expertise for Parkinson & Movement Disorders was supported by a center of excellence grant by the Parkinson's Foundation. IK and HBK have no interests to declare.

The authors declare that none of the above interests present a conflict with the present study.
