# Supplementary material for: Serum aromatic l-amino acid decarboxylase activity as a biomarker for prodromal and manifest Parkinson's disease
Source: eBioMedicine. 2026 Jul 9;130:106354. doi: 10.1016/j.ebiom.2026.106354 (PMC13380500; doi:10.1016/j.ebiom.2026.106354)
Supplement: Supplementary Material [file mmc1.pdf]

Supplementary Material

Table of contents

Supplementary Table 1	Controls cohort……………………………………………………………2
Supplementary Table 2	Diagnostic performance of various serum AADC enzyme activity values as a marker for (unmedicated, clinically manifest) Parkinson’s disease………..3
Supplementary Table 3	Regression table for dopaminergic medication and disease duration as predictors of serum AADC enzyme activity…………………………………4
Supplementary Table 4	Regression table for levodopa/PDI use and disease duration as predictors of serum AADC enzyme activity………………………………………………4
Supplementary Table 5	Regression table for COMT inhibitor use and disease duration as predictors of serum AADC enzyme activity……………………………………………4
Supplementary Table 6	Regression table for levodopa-equivalent daily dose (LEDD), disease duration and sex as predictors of serum AADC enzyme activity……………4
Supplementary Table 7	Regression table for serum AADC enzyme activity, disease duration, levodopa-equivalent daily dose (LEDD) and sex as predictors of daily OFF duration………………………………………………………………… 5
Supplementary Table 8	Regression table for serum AADC enzyme activity, disease duration, levodopa-equivalent daily dose (LEDD) and sex as predictors of MDS-UPDRS item 4.5 score (complexity of fluctuations)…………………………5
Supplementary Table 9	Regression table for disease duration, sex, use of levodopa/PDI and levodopa-equivalent daily dose (LEDD) as predictors of serum AADC enzyme activity…………………………………………………………… 6
Supplementary Figure 1	Serum AADC enzyme activity after same-day processing vs. delayed processing………………………………………………………………..6
Supplementary Figure 2	Calibration plot of serum AADC enzyme activity as a diagnostic for probable prodromal Parkinson’s disease (AG4) vs. controls (AG5)……….. 7
Supplementary Figure 3	Calibration plot of serum AADC enzyme activity as a diagnostic for early unmedicated Parkinson’s disease (AG2) vs. controls (AG5)……………….. 8
Supplementary Figure 4	AADC enzyme activity per type of medication used………………………..9
Supplementary Figure 5	AADC enzyme activity vs. complexity of motor fluctuations……………...10
Supplementary Box 1	Cohort descriptions……………………………………………………… 10
Supplementary Box 2	AADC assay………………………………………………………………11

Supplementary Table 1 Controls cohort

Diagnosis	Known AADC-activity-influencing factors?	Serum AADC enzyme activity with Z-value >3 or <-3?
Chronic (peri)orbital pain	No	No
Polyneuropathy	No	No
Basilar arteriopathy	No	No
Polyradiculopathy	No	No
Idiopathic intracranial hypertension	No	No
Lumbosacral plexitis (neoplastic etiology)	No	No
Radiculopathy	No	No
Neuroinflammatory laesion medulla oblongata	No	No
Intracranial hypertension e.c.i.	No	No
Hemipyramidal syndrome	No	No
Subacute cognitive deficit caused by dexamethasone	No	No
Radiculopathy	No	No
Muscle weakness after CABG	No	No
Psychiatric disease	Dopamine receptor blocker use	Yes (96·8 mU/L)
Primary lateral sclerosis (PLS)	No	No
Myelopathy e.c.i.	No	No
Pain e.c.i.	No	No
Amyotrophic lateral sclerosis (ALS)	No	No
Normal-pressure hydrocephalus	No	No
Polyradiculopathy	No	No
Diabetic polyradiculopathy and plexopathy	No	No
VZV encephalitis	No	No
Unexplained pain	No	No
Cognitive deficits e.c.i.	No	Yes (96·2 mU/L)
Opticopathy e.c.i.	No	No
Progressive spinal muscular atrophy (PSMA)	No	No
Radiculopathy	No	No
Normal-pressure hydrocephalus	No	No
Lymphoma	No	No
Abducens nerve palsy	No	No
Essential tremor	No	No
Intracerebral haemorrhage	No	No
Myelopathy	No	No
Hypoglossal neuropathy	No	No
Lumbosacral plexopathy	No	No
Cerebral metastasis of melanoma	No	No
Progressive spinal muscular atrophy (PSMA)	No	No
Bipyramidal syndrome e.c.i.	No	No
Radiculopathy	No	No
Tolosa-Hunt syndrome	No	No
Chronic inflammatory demyelinating polyneuropathy (CIDP)	No	No
Abducens nerve palsy	No	No
Facial pain	No	No
Leg pain	No	No
Intracranial hypertension	No	No
Radiculopathy	No	No
Small-fibre neuropathy (SCN10A mutation)	No	No
Facial sensory complaints e.c.i.	No	No
Hyperventilation	No	No
Leg pain	No	No
Benign thunderclap headache	No	No
White matter lesions e.c.i.	No	No
Cognitive deficits e.c.i.	No	No
(Para)neoplastic leg pain	No	No
Muscle and joint pain e.c.i.	No	No
Rule out neurosyphilis	No	No

Chronic daily headache	No	No
Acute headache e.c.i.	No	No
Epileptic seizure	No	No
Depression and subjective cognitive deficits, insufficient clues for neurodegenerative condition	No	Yes (103·5 mU/L)
Polyneuropathy	Dopamine receptor blocker use	No
Cognitive deficits, probably drug-induced	No	No
Headache	No	No
Post-transplant lymphoma (no CNS lymphoma)	Renal insufficiency and significant liver test abnormalities	No
Chorea e.c.i.	No	No
Chronic bilateral opticopathy e.c.i.	No	No
Lymphoma with CNS localisation	Renal insufficiency and significant liver test abnormalities	No
Headache	No	No
Cerebral infarction	Dopamine receptor blocker use	No
Headache	No	No
Normal-pressure hydrocephalus	No	No
Cocaine-induced leukencephalopathy	No	No
Brachial plexopathy	Dexamethasone and dopamine receptor blocker use	No
Radiculopathy	No	No

AADC: aromatic *L*-amino acid decarboxylase, CABG: coronary artery bypass graft, CNS: central nervous system. Comparison of number of outliers in controls with vs. without known AADC-influencing factors: Fisher's exact test *P*=0·23.

Supplementary Table 2 Diagnostic performance of various serum AADC enzyme activity values as a marker for (unmedicated, clinically manifest) Parkinson's disease

Serum AADC enzyme activity (mU/L)	Sensitivity	Specificity	Positive predictive value (PPV)	Negative predictive value (NPV)	Accuracy	Miscellaneous
14·86	100%	4·1%	61·4%	100%	62·6%	
28·30	93·1%	31·1%	67·9%	74·2%	68·9%	Max F-score (0·785)
35·06	76·7%	50%	70·6%	57.8%	66·3%	
35·90	75·0%	58·1%	73·7%	59·7%	68·4%	Max Youden's J (0·331)
43·56	55·2%	75·7%	78·0%	51·9%	63·2%	
50(·07)	32·8%	79·7%	71·7%	43·1%	51·1%	
52·00	28·4%	81·1%	70·2%	42·0%	48·9%	
81·25	6·9%	95·9%	72·7%	39·7%	41·6%	
113·65	3·4%	100%	100%	39·8%	41·1%	

AADC: aromatic *L*-amino acid decarboxylase, PD: Parkinson's disease

Supplementary Table 3 Regression table for dopaminergic medication and disease duration as predictors of serum AADC enzyme activity

Variable	Slope (β)	SE	95% CI		*P*
			LL	*UL*	
Intercept	39·09	1·92	35·44	42·98	<0·001
Use of dopaminergic medication	53·50	4·63	43·98	62·02	<0·001
Disease duration (months)	0·49	0·09	0·32	0·69	<0·001

Regression table for linear regression with serum AADC enzyme activity (mU/L) as the dependent variable, in medicated + unmedicated early Parkinson's disease (AG1+AG2). 95%CI is for a bootstrapped analysis with 1.000 samples, with outliers trimmed.
SE: standard error, CI: confidence interval, LL: lower limit, UL: upper limit.

Supplementary Table 4 Regression table for levodopa/PDI use and disease duration as predictors of serum AADC enzyme activity

Variable	Slope (β)	SE	95% CI		*P*
			LL	*UL*	
Intercept	40·04	2·09	36·16	44·10	<0·001
Use of levodopa/PDI	56·81	5·27	45·84	66·73	<0·001
Disease duration (months)	0·42	0·12	0·20	0·67	<0·001

Regression table for linear regression with serum AADC enzyme activity (mU/L) as the dependent variable, in medicated + unmedicated early Parkinson's disease (AG1+AG2). 95%CI is for a bootstrapped analysis with 1.000 samples, with outliers trimmed.
PDI: peripheral decarboxylase inhibitor, SE: standard error, CI: confidence interval, LL: lower limit, UL: upper limit.

Supplementary Table 5 Regression table for COMT inhibitor use and disease duration as predictors of serum AADC enzyme activity

Variable	Slope (β)	SE	95% CI		*P*
			LL	*UL*	
Intercept	89·07	7·67	74·12	105·13	<0·001
Use of COMT inhibitor	28·08	12·43	4·00	51·42	0·01
Disease duration (months)	0·58	0·15	0·27	0·88	<0·001

Regression table for linear regression with serum AADC enzyme activity (mU/L) as the dependent variable, in people with early Parkinson's disease who use levodopa/PDI (AG1, filtered for levodopa/PDI users). 95%CI is for a bootstrapped analysis with 1.000 samples, with outliers trimmed.
COMT: catechol-*O*-methyltransferase, PDI: peripheral decarboxylase inhibitor, SE: standard error, CI: confidence interval, LL: lower limit, UL: upper limit.

Supplementary Table 6 Regression table for levodopa-equivalent daily dose (LEDD), disease duration and sex as predictors of serum AADC enzyme activity

Variable	Slope (β)	SE	95% CI		*P*
			LL	*UL*	
Intercept	69·79	6·60	56·63	82·19	<0·001
LEDD (mg/24h)	0·03	0·01	0·01	0·04	<0·001
Disease duration (months)	0·48	0·12	0·24	0·71	<0·001
Female sex	14·78	4·57	5·69	23·74	<0·001

Regression table for linear regression with serum AADC enzyme activity (mU/L) as the dependent variable, in early medicated Parkinson's disease (AG1). 95%CI is for a bootstrapped analysis with 1.000 samples, with outliers trimmed.
LEDD: levodopa-equivalent daily dose, SE: standard error, CI: confidence interval, LL: lower limit, UL: upper limit.

Supplementary Table 7 Regression table for serum AADC enzyme activity, disease duration, levodopa-equivalent daily dose (LEDD) and sex as predictors of daily OFF duration

Daily OFF duration	Predictor variable	B	SE	OR	95% CI of OR		*P*
					LB	*UB*	
1 hour	Intercept	-2·487	0·549				<0·001
	Serum AADC activity (mU/L)	0·004	0·003	1·004	0·998	1·010	0·224
	Disease duration (months)	0·001	0·008	1·001	0·985	1·017	0·915
	LEDD (mg/24h)	0·001	0·000	1·001	1·001	1·002	0·002
	Female sex	0·386	0·300	1·471	0·817	2·648	0·199
2 hours	Intercept	-3·269	0·638				<0·001
	Serum AADC activity (mU/L)	0·005	0·003	1·005	0·998	1·012	0·161
	Disease duration (months)	0·002	0·009	1·002	0·984	1·020	0·827
	LEDD (mg/24h)	0·002	0·001	1·002	1·001	1·003	<0·001
	Female sex	0·304	0·344	1·355	0·690	2·660	0·378
3 hours	Intercept	-4·044	0·856				<0·001
	Serum AADC activity (mU/L)	0·008	0·004	1·008	1·000	1·017	0·058
	Disease duration (months)	0·010	0·012	1·010	0·986	1·034	0·427
	LEDD (mg/24h)	0·001	0·001	1·001	0·999	1·002	0·296
	Female sex	0·246	0·453	1·279	0·526	3·109	0·587
≥4 hours	Intercept	-3·771	0·669				<0·001
	Serum AADC activity (mU/L)	-0·001	0·004	0·999	0·991	1·007	0·799
	Disease duration (months)	0·010	0·010	1·011	0·991	1·030	0·283
	LEDD (mg/24h)	0·003	0·001	1·003	1·002	1·004	<0·001
	Female sex	0·210	0·374	1·233	0·592	2·568	0·575

Regression table for multinomial logistic regression with daily OFF duration as the dependent variable, in early medicated Parkinson's disease (AG1). Zero (0) OFF hours is the reference category. 95%CI is with outliers trimmed.
B: unstandardised regression weight, LEDD: levodopa-equivalent daily dose, SE: standard error, OR: odds ratio, CI: confidence interval, LB: lower bound, UB: upper bound.

Supplementary Table 8 Regression table for serum AADC enzyme activity, disease duration, levodopa-equivalent daily dose (LEDD) and sex as predictors of MDS-UPDRS item 4.5 score (complexity of fluctuations)

MDS-UPDRS item 4.5 score	Predictor variable	B	SE	OR	95% CI of OR		*P*
					LB	*UB*	
1	Intercept	-2·245	0·417				<0·001
	Serum AADC activity (mU/L)	0·004	0·002	1·004	1·000	1·009	0·065
	Disease duration (months)	0·008	0·006	1·008	0·996	1·020	0·177
	LEDD (mg/24h)	0·001	0·000	1·001	1·001	1·002	<0·001
	Female sex	0·414	0·224	1·513	0·976	2·345	0·064
2	Intercept	-3·070	0·635				<0·001
	Serum AADC activity (mU/L)	0·002	0·004	1·002	0·994	1·009	0·669
	Disease duration (months)	0·001	0·009	1·001	0·983	1·019	0·956
	LEDD (mg/24h)	0·002	0·000	1·002	1·001	1·003	<0·001
	Female sex	0·126	0·351	1·134	0·570	2·256	0·720
3	Intercept	-4·928	0·907				<0·001
	Serum AADC activity (mU/L)	0·001	0·005	1·001	0·992	1·010	0·834
	Disease duration (months)	0·025	0·012	1·025	1·001	1·050	0·045
	LEDD (mg/24h)	0·001	0·001	1·001	1·000	1·003	0·018
	Female sex	0·691	0·448	1·995	0·829	4·801	0·123
≥4	Intercept	-5·374	1·356				<0·001
	Serum AADC activity (mU/L)	0·006	0·007	1·006	0·993	1·019	0·353
	Disease duration (months)	0·019	0·018	1·019	0·983	1·056	0·313

LEDD (mg/24h)	0·001	0·001	1·001	0·999	1·003	0·172
Female sex	-1·539	1·080	0·215	0·026	1·783	0·154

Regression table for multinomial logistic regression with MDS-UPDRS item 4.5 score (complexity of fluctuations) as the dependent variable, in early medicated Parkinson's disease (AG1). A score of zero (0) is the reference category. 95%CI is with outliers trimmed.
MDS-UPDRS: Movement Disorder Society-sponsored revision of the Unified Parkinson's Disease Rating Scale, B: unstandardised regression weight, LEDD: levodopa-equivalent daily dose, SE: standard error, OR: odds ratio, CI: confidence interval, LB: lower bound, UB: upper bound.

Supplementary Table 9 Regression table for disease duration, sex, use of levodopa/PDI and levodopa-equivalent daily dose (LEDD) as predictors of serum AADC enzyme activity

**Variable**	**Slope (β)**	**SE**	**95% CI**		***P***
			LL	*UL*	
Intercept	27·25	11·44	4·62	49·51	0·02
Disease duration (months)	0·47	0·12	0·24	0·72	<0·001
Female sex	15·54	4·29	7·06	23·42	<0·001
Use of levodopa/PDI	48·30	10·42	26·01	67·45	<0·001
LEDD (mg/24h)	0·02	0·01	0·01	0·03	0·01

Regression table for linear regression with serum AADC enzyme activity (mU/L) as the dependent variable, in early medicated + unmedicated Parkinson's disease (AG1+AG2). 95%CI is for a bootstrapped analysis with 1.000 samples, with outliers trimmed.
PDI: peripheral decarboxylase inhibitor, LEDD: levodopa-equivalent daily dose, SE: standard error, CI: confidence interval, LL: lower limit, UL: upper limit.

Supplementary Figure 1 Serum AADC enzyme activity after same-day processing vs. delayed processing

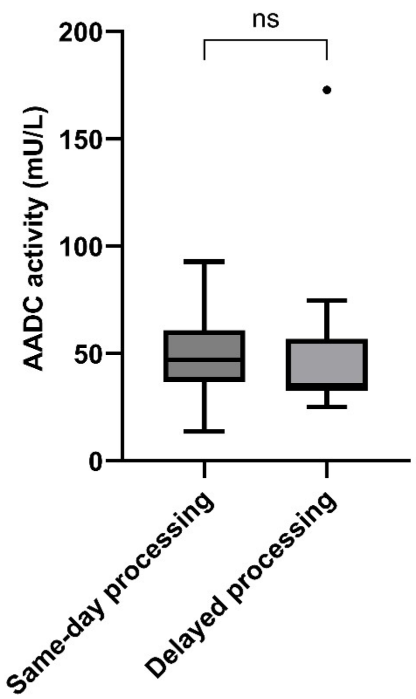

Box plots of (non-adjusted) serum AADC enzyme activity in the Luxembourg RBD Study cohort, grouped by sample processing on the day of blood collection ('same-day processing', *n*=33) versus processing on the day after ('delayed processing', *n*=17). Significance marker is for Mann-Whitney U. ns: non-significant.

Supplementary Figure 2 Calibration plot of serum AADC enzyme activity as a diagnostic for probable prodromal Parkinson's disease (AG4) vs. controls (AG5)

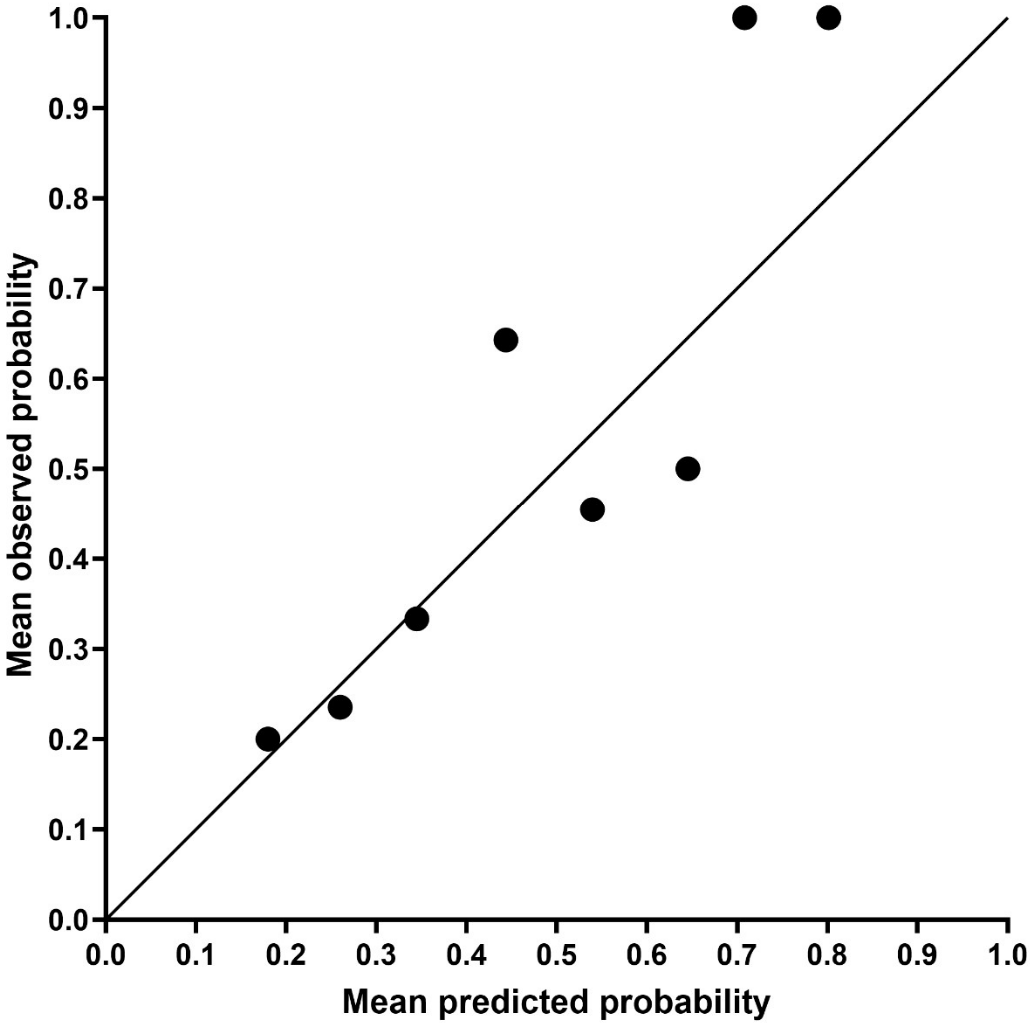

The x-axis displays the predicted probability of the diagnosis 'probable prodromal Parkinson's disease', as determined using binary logistic regression with serum AADC enzyme activity as the independent variable and diagnosis (1: probable prodromal Parkinson's disease, 0: control) as the dependent variable, the value averaged per decile. The y-axis displays the observed probability of these diagnoses in the combined group of probable prodromal Parkinson's disease (AG4, *n*=43) and controls (AG5, *n*=74), averaged per predicted decile.

Supplementary Figure 3 Calibration plot of serum AADC enzyme activity as a diagnostic for early unmedicated Parkinson's disease (AG2) vs. controls (AG5)

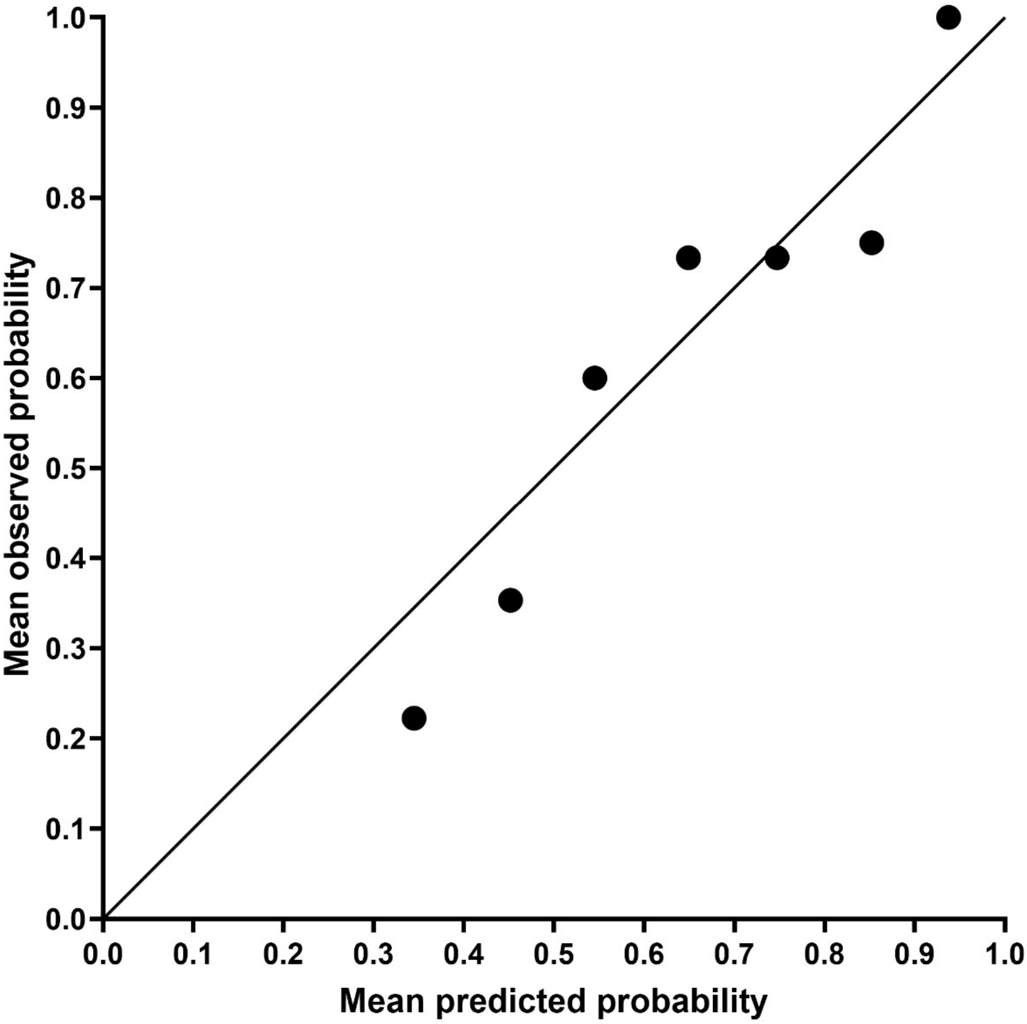

The x-axis displays the predicted probability of the diagnosis 'early (unmedicated) Parkinson's disease', as determined using binary logistic regression with serum AADC enzyme activity as the independent variable and diagnosis (1: early unmedicated Parkinson's disease, 0: control) as the dependent variable, the value averaged per decile. The y-axis displays the observed probability of these diagnoses in the combined group of early unmedicated Parkinson's disease (AG2, *n*=116) and controls (AG5, *n*=74), averaged per predicted decile.

Supplementary Figure 4 AADC enzyme activity per type of medication used

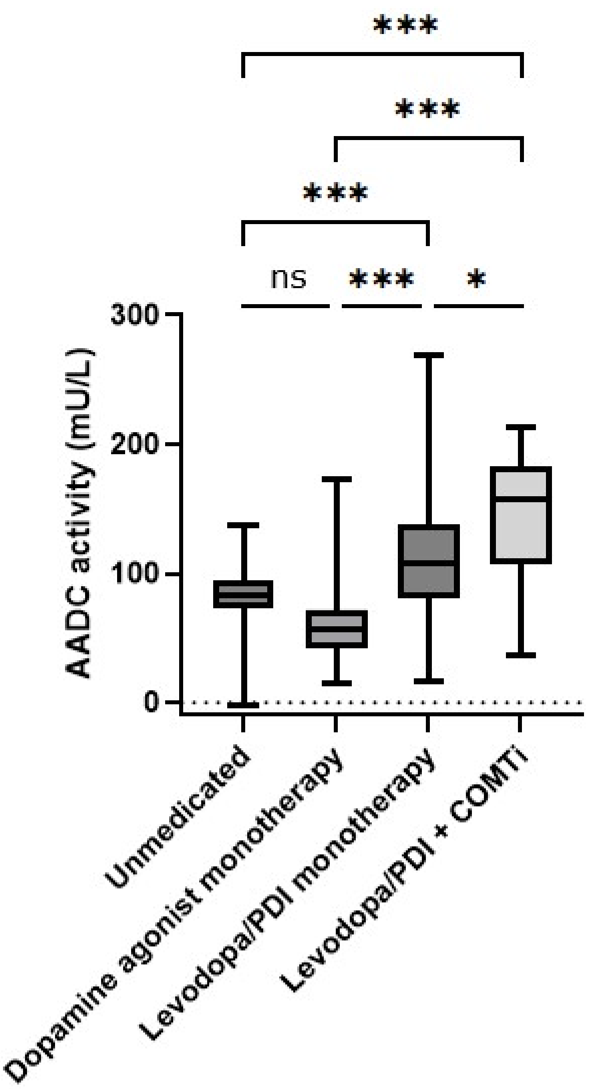

Box plots of serum AADC enzyme activity in early Parkinson's disease (AG1 and AG2), grouped by type of dopaminergic medication used (unmedicated *n*=112, dopamine agonist monotherapy *n*=10, levodopa/PDI monotherapy *n*=255, levodopa/PDI + COMTi *n*=20). AADC enzyme activity is adjusted for disease duration.

COMTi: catechol-*O*-methyltransferase inhibitor, PDI: peripheral decarboxylase inhibitor.

Significance markers are for Kruskal-Wallis test with post-hoc inter-group comparisons. Significance, after Benjamini-Hochberg adjustment for multiple testing, is noted by the number of asterisks: * *P* <0·05, ** *P* <0·01, *** *P* <0·001, ns non-significant.

Supplementary Figure 5 AADC enzyme activity vs. complexity of motor fluctuations

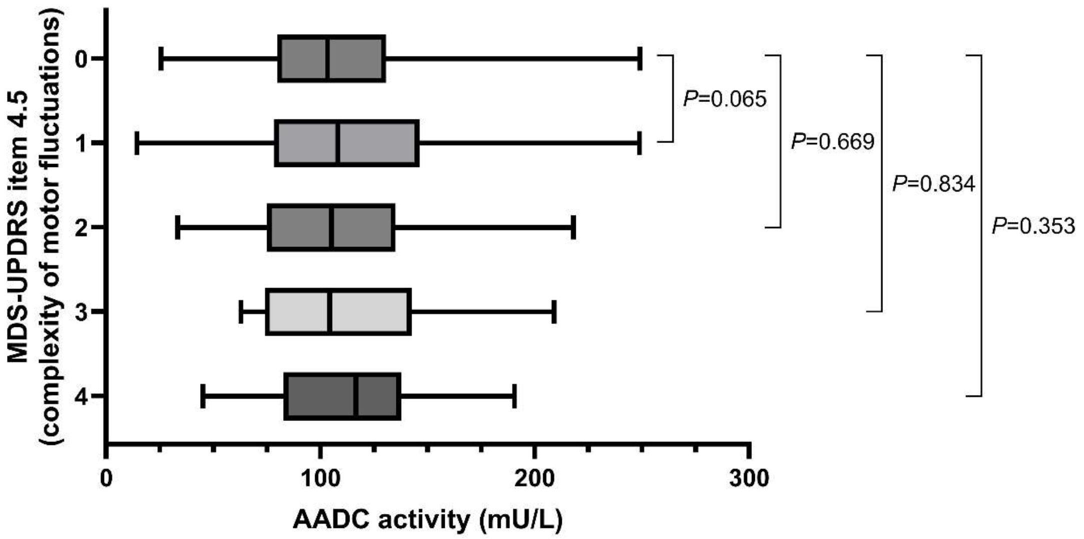

Box plots of serum AADC enzyme activity in medicated early Parkinson's disease (AG1), grouped by the score on item 4.5 of the Movement Disorder Society-sponsored revision of the Unified Parkinson's Disease Rating Scale part IV (complexity of motor fluctuations), on which a higher number denotes a higher complexity of fluctuations (i.e. more unpredictable). Score 0 *n*=210, score 1 *n*=179, score 2 *n*=47, score 3 *n*=24, score 4 *n*=9. AADC enzyme activity is adjusted for disease duration (mean), sex (mode) and LEDD (mean). *P* values are for multinomial logistic regression.

Supplementary Box 1 Cohort descriptions

(i) Personalized Parkinson Project

- *n*=516, Parkinson's disease, medicated as well as unmedicated
- Inclusion: PD with ≤5 years of disease duration since diagnosis by a neurologist
- Exclusion: pregnancy or breastfeeding, comorbidities that would hamper interpretation of parkinsonian disability, MRI contraindications such as ferromagnetic implants
- Three annual visits between 2017 and 2023 at Radboudumc, Nijmegen, the Netherlands
- Type of collected data: demographic data, exposome data, clinical tests (including MDS-UPDRS and cognitive testing), biosamples (blood, faeces, cerebrospinal fluid, MRI), wearable data (smartwatch)

(ii) Personalized Parkinson Project de novo

- *n*=102, unmedicated PD
- Inclusion: PD with ≤2 years of disease duration since diagnosis by a neurologist, never treated with dopaminergic drugs (including mucuna pruriens) and not expected to start treatment within 52 weeks
- Exclusion: comorbidities that would hamper interpretation of parkinsonian disability
- Three annual visits between 2020 and 2025 at Radboudumc, Nijmegen, the Netherlands
- Type of collected data: demographic data, exposome data, clinical tests (including MDS-UPDRS and cognitive testing), biosamples (blood), wearable data (smartwatch)

(iii) AADCTDC study

- *n*=48
- Inclusion: PD with ≥5 years of disease duration since diagnosis by a neurologist
- Exclusion: comorbidities that would hamper interpretation of parkinsonian disability, contra-indications to levodopa/benserazide, antibiotic use up to 1 year prior to study participation, use of AADC-influencing drugs, gastro-intestinal comorbidity
- One visit between 2023 and 2024 at Radboudumc, Nijmegen, the Netherlands
- Type of collected data: demographic data, exposome data, clinical tests (including MDS-UPDRS), biosamples (blood, faeces, urine)

(iv) Luxembourg RBD Study

- *n*=54
- Inclusion: PSG-confirmed RBD. age 55 to 75 years with enrichment recruitment strategy identifying individuals with highest probability of RBD based on REM-Sleep Behaviour Disorder Screening Questionnaire (RBDSQ) and on-site evaluation by neurologist and neuropsychologist. The individuals with highest clinical plausibility of RBD in absence of parkinsonism and/or dementia and presenting a hyposmia as defined by a smell test (BSIT-A) were validated by PSG as defined by the diagnostic criteria of RBD by International Classification of Sleep Disorders, Third Edition (ICSD-3)
- Exclusion: clinically manifest Parkinson's disease
- Annual visits between years 2021 and 2024 at Centre Hospitalier de Luxemborg, Belair, Luxembourg
- Type of collected data: demographic data, sleep data (questionnaires and polysomnography), smell tests (BSIT, Sniffin'Sticks), clinical tests (including MDS-UPDRS, cognitive tests), vision tests, biosamples (blood, urine, saliva, skin biopsy, cerebrospinal fluid, faeces)

(v) Controls

- *n*=74
- Inclusion: presence of leftover serum sample after venipuncture for clinical purposes, no objection to the reuse of the samples for research purposes
- Exclusion: diagnosis of PD or another diagnosis associated with dopaminergic neurodegeneration, use of levodopa, impaired renal function, impaired hepatic function, use of medication with dopamine receptor blocking action
- Type of collected data: demographic data, diagnosis and medication use (from electronic health record), biosamples (blood)
- Blood samples collected between 2012 – 2022 at Radboudumc, Nijmegen, the Netherlands

Supplementary Box 2 AADC assay

After venipuncture and collection of blood in a serum tube (with clot activator, no gel, no protease inhibitor), coagulation was allowed for at least 30 minutes (Luxembourg) or 60-120 minutes (Radboudumc), then after a maximum of 4 hours (preferably <2 hours) centrifugation was carried out at 2000g for 10 minutes at room temperature. A minority of samples (*n*=17) were processed the day after blood withdrawal. Serum was stored in 220 µL (Luxembourg) or 0.5 mL (Radboudumc) aliquots in 2 mL polypropylene cryovials at -80 °C.
For the enzymatic reaction, 50 µL of serum was added to 355 µL phosphate buffer (167 mM, pH 7·0) containing 39 mmol/L DL-dithiothreitol, 0·167 mmol EDTA and 75 µL pyridoxal-5 phosphate (0·7 mM). Samples were pre-incubated for 2 hours at 37 °C while shaking at 225 rpm. 130 µL of levodopa substrate (12·5 mM) was added and samples were incubated for 1 hour at 37 °C while shaking at 225 rpm, after which the reaction was stopped by addition of formic acid (30 µL, 5% HCOOH). After the addition of dopamine-d3 as an internal standard (cat. no. D533785, Toronto Research Chemicals, Canada) the reaction buffer was passed through a 30 kDa centrifugal filter (Amicon® Ultra 0·5 mL 30 kD centrifugal filter regenerated cellulose 30,000 MWCO, Millipore) to remove protein (15 min. 14,000 × *g,* 15 °C). One µl of the 30 kD filtrate was diluted with 100 µl of MilliQ and propionylated by adding 50 µl of 10% pyridine in acetonitrile and 50 µl of 4% propionic anhydride in acetonitrile. After vortexing, the solution was dried using nitrogen at 40 °C and redissolved in 30 µl of 20% acetonitrile in MQ. Using this procedure, dopamine (and its stable isotope analogue) will be triple propionylated. Two µl were injected into the UPLC-MS/MS system which consists of a Waters I-Class Acquity fitted with a GL Sciences Inert Sustain AQ C18 column (2·1*100 mm dp 1·9 µ) connected to a Waters Xevo TQSµ mass spectrometer. The column was run at 400 µl/min in gradient mode at 50 °C using 0·5% acetic acid in water and acetonitrile (initial conditions 20% 0·5% acetic acid in water, gradient: 10% acetonitrile per minute). The column flow was directed to a Xevo TQSµ fitted with an electrospray ionisation probe operated in positive mode at unit resolution. The capillary voltage was set at 0·7 kV. The temperature settings for the source and ion block were set at 550 °C and 150 °C, respectively. As a drying gas, nitrogen was used at a flow rate of 1200 L/h. The cone gas flow was set at 100 L/h. The cone voltage was set at 30 V and the collision cell was operated with argon as the collision gas at a pressure of 0·35 Pa and a voltage of 30 eV. Quantification was done by comparison of the generated area response using the stable isotope as internal standard.
